# Assessing fatty acid oxidation flux in rodent cardiomyocyte models

**DOI:** 10.1038/s41598-018-19478-9

**Published:** 2018-01-24

**Authors:** M. Rech, J. J. F. P. Luiken, J. F. C. Glatz, M. van Bilsen, B. Schroen, M. Nabben

**Affiliations:** 1 Department of Cardiology, CARIM School For Cardiovascular Diseases, Maastricht University, Maastricht, The Netherlands; 2 Department of Genetics and Cell Biology, CARIM school for cardiovascular diseases, Maastricht University, Maastricht, The Netherlands

## Abstract

The healthy adult heart primarily relies on fatty acid oxidation (FAO) for energy production but instantaneously adapts its substrate preference in response to physiological or pathological challenges. Accurate FAO measurements are crucial to investigate early metabolic (mal)adaptations. While measurements in intact cardiomyocytes offer greater physiological relevance, current FAO protocols mainly employ cell-free systems and/or require expensive equipment. Here, we present an easy-to-use, inexpensive, and sensitive method to measure, compare and modulate FAO in various cardiomyocyte models. Basal FAO was 2-fold higher in fresh versus cultured adult rat cardiomyocytes (aRCM), while OXPHOS protein levels were maintained. Basal FAO was higher in cultured (3-fold) and fresh (8-fold) aRCM, versus widely used neonatal rat cardiomyocytes (nRCM) and mouse HL1 cardiomyocytes. Moreover, we utilized chemical and pharmacological treatments in order to modulate the FAO flux at different cellular signalling levels. Our data indicate that caution should be taken when studying metabolism in nRCM and HL1 cell models, as these display significantly lower FAO than aRCM. Accurate FAO measurement in cultured aRCM opens new avenues for studying the complex cardiomyocyte metabolic responses to mechanical, nutritional, pharmacological, and genetic manipulations.

## Introduction

Long-chain fatty acids (FA) constitute the main energy source of the heart under aerobic conditions and are the preferred substrate for mitochondrial oxidation at rest. Upon physiological and pathological challenges the heart is flexible to shift its substrate preference^1^. For instance, the development of heart failure usually is accompanied by an early shift towards a greater reliance on glycolytic substrates and a concomitant suppression of fatty acid oxidation (FAO). Conversely, among the early changes in the hearts of diabetic/obese patients is the almost exclusive reliance on FAO and the loss of ability to switch between substrates. Since changes in substrate preference often reflect cardiac pathologies, metabolic (mal)adaptation of cardiomyocytes has generated considerable research interest.

*In vitro* and *ex vivo* models are essential means to investigate mechanisms of disease development and progression and provide efficient platforms for drug screening. Current protocols for measuring FAO often use isolated mitochondria or permeabilized cells and provide measures of mitochondrial FAO capacity under near optimal conditions. In intact cardiomyocytes, however, FAO flux depends on expression and functioning of the proteins involved in cellular FA uptake, mitochondrial import and β-oxidation of FA, and on tricarboxylic acid (TCA) cycle and respiratory chain activities under the prevailing (intra)cellular conditions. Therefore, the measurement of FAO flux in intact living cells is a preferred tool to assess cardiac metabolic state. Current measurements of FAO in intact cells are often estimations based on oxygen consumption rate. Besides that these methods require expensive equipment, it is hard to control which specific substrates are oxidized by the mitochondria. Moreover, while the medium can be supplemented with various substrates, cells can store and oxidize endogenous pools of substrates, making it difficult to determine which specific metabolic pathways are fuelling respiration. A direct and relatively inexpensive method to measure FAO is through the use of radiolabeled substrates. This allows for complete oxidation and precise determination of the metabolic fate of the specific substrate.

An established protocol for measurement of FAO flux in cultured adult rat cardiomyocytes (aRCM) is currently missing. Here, we set-up a protocol with [1-^14^C]-labeled palmitate for use in cultured primary aRCM to allow for accurate measurement of metabolic adaptations to mechanical, nutritional, pharmacological, and genetic manipulations, thereby opening a new avenue for the study of the complex cardiomyocyte metabolism. We used freshly isolated aRCM, in which FAO kinetics were previously studied in detail by Luiken *et al*.^2^, as reference point. For comparison we measured FAO flux in intact fresh aRCM, cultured aRCM, and two commonly used cardiomyocyte models, i.e., neonatal rat cardiomyocytes (nRCM) and HL1 cells from the AT-1 mouse atrial myocyte tumor lineage. Additionally, we characterized the effect on FAO of several compounds known to modulate FAO. Furthermore, we studied the sensitivity of our protocol for the measurement of FAO flux in the cardiomyocytes of an *in vivo* model of pre-diabetes.

## Materials and Methods

### Cardiomyocyte isolation and culture

All experiments were performed in accordance with the approved guidelines and regulations and complied with the principles of laboratory animal care. All protocols were approved by the Maastricht University animal ethics committee. Details of animals, chemicals, media and notes are provided in the supplementary information. aRCM were isolated via Langendorff perfusion as previously described^2^, seeded (2*10^5^ cells/well) in laminin coated plates, and cultured in aRCM medium. nRCM were isolated as previously described^3^, seeded (5*10^5^ cells/well) in gelatin type B coated plates and cultured in nRCM medium. HL1 cells were seeded (3*10^5^ cells/well) and cultured as previously described^4^.

Prior to FAO flux measurement, cells were cultured in 12-well plates in 1 mL of the respective medium for 48 hours. For comparison of cell types, after culturing, the medium of all cell types was switched into aRCM medium. Cells were detached by gentle scraping (supplementary note 1). Cell quality and morphology were checked microscopically and viability was assessed by trypan blue staining (supplementary note 2). For western blot and RNA analysis samples were lysed in RIPA or lysis buffer, respectively, and stored at −80 C until use. Details on western blot and RNA analysis are described in the supplementary information.

### Fatty acid oxidation assay

**Figure 1:**
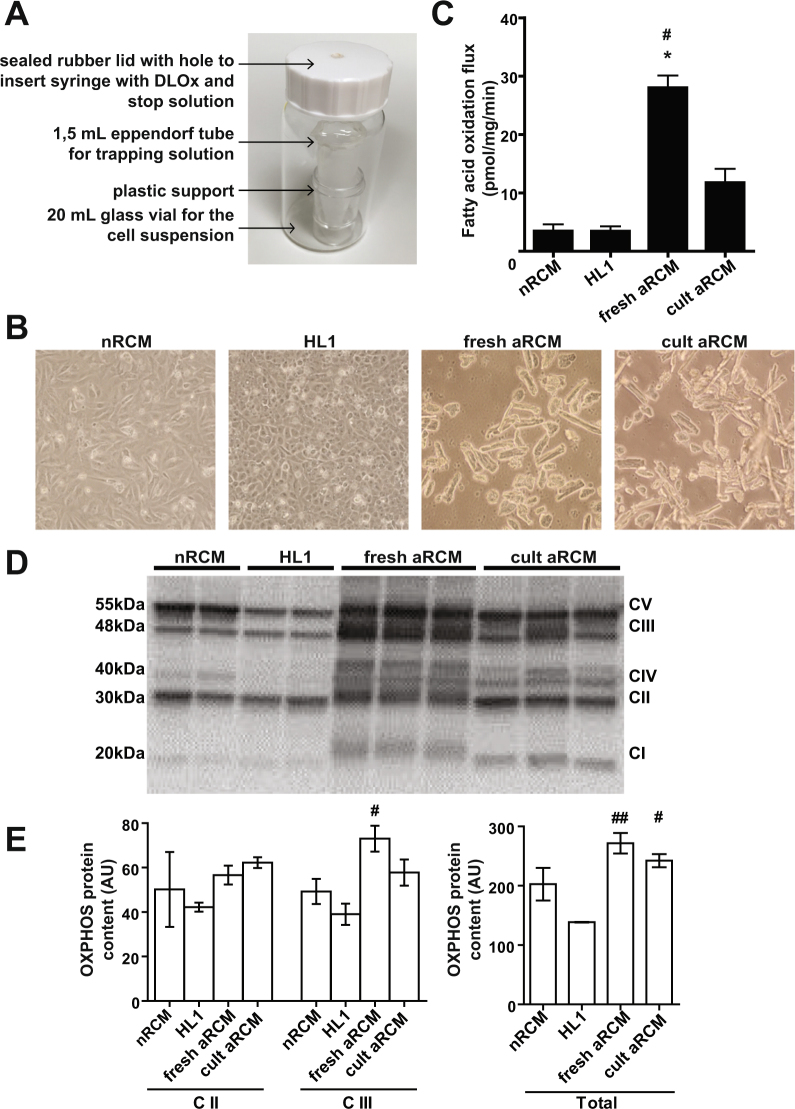
Application of the FAO flux protocol for comparison of cardiomyocyte-types (**A**) Assay setup; (**B**) Inverted microscope images of cardiomyocyte-types, 20× magnification; (**C**) Quantification of FAO flux in neonatal rat cardiomyocytes (nRCM), HL1 cardiomyocytes (HL1), adult rat cardiomyocytes (aRCM) fresh and cultured (n = 4; 3; 17; 3); (**D**) and (**E**) Representative western blot and quantification of OXPHOS protein; the full-length blot is presented in Supplementary Figure S1. Data are presented as mean ± SEM; *p < 0.01 vs nRCM, ^#^p < 0.01 vs HL1.

The following solutions were prepared prior to performing the assay: *Stock [1-*^14^*C] palmitate label*: as described previously^5^; *Wash Solution:* Medium M199, containing 0.9% of BSA; *Day Label Oxidation Solution* (DLOx): *Wash Solution* and ^14^C-labeled palmitate-albumin complex in a ratio of 0.23: 0.77, *v/v*, and pre-heated at 37 °C; *Trapping Solution*: mixing ethanolamine and ethylene glycol in a ratio of 1:2, *v/v*; *Stop Solution*: 3 M of perchloric acid. After scraping, cardiomyocytes were transferred into a 20-mL glass vial containing an Eppendorf tube with trapping solution (Fig. 1A). The wells were washed with 1 mL *Wash Solution* to collect any remaining cells. Subsequently, the cell suspension (2 mL in total) was gassed with carbogen (95% O_2_: 5% CO_2_) and the vials were immediately sealed with a rubber cap and lid. Subsequently, 0.5 mL of the pre-heated DLOx was added to the cells with a 1 mL syringe via the sealed rubber lid (final palmitate and BSA concentrations 90 and 120 μM, respectively) (supplementary note 3). Upon injection of the DLOx, samples were placed for 30 min at 37 °C in a water bath with gentle shaking. The oxidation reaction was terminated by adding 0.4 mL *Stop Solution* to the cell suspension via the sealed rubber lid. The glass vials with cells were placed at 4 °C for at least 4 hours to allow trapping of ^14^CO_2_. Afterwards, the trapping solution (containing radioactive ^14^CO_2_) was transferred into a glass beta counter vial containing 10 mL Optifluor. Cells were lysed and total protein concentration was assessed. Radioactivity was measured by liquid scintillation counting using a 5 min counting protocol (Quench Indicator: tSIE/AEC). FAO fluxes were expressed as nmol/mg cell protein per min.

Stimulation protocol: Oligomycin (final concentration 5 µM) or rotenone (final concentration 3 µM) were added to the cell suspensions before placing the samples in the water bath, allowing a total of 30 min incubation. Peroxisome proliferator activator receptor (PPAR)α ligand WY-14,643 (final concentration 10 μM) was added to the cardiomyocytes already in the culture plates for 48 hours. In each experiment we included a so-called zero time control (ZTC) condition as a negative control (supplementary note 4).

### Statistical analysis

Data are presented as average ± SEM. Comparisons between 2 groups were performed with the 2-tailed Student’s *t*-test for Gaussian data. For comparisons of >2 groups, one-way or two-way ANOVA was used followed by post hoc testing with Bonferroni correction. Values of p < 0.05 were considered statistically significant.

### Data availability

All data generated and analysed during this study are included in this article.

## Results and Discussion

### Comparison of fatty acid oxidation flux among distinct cardiomyocyte models

To analyze FAO fluxes in commonly used cardiomyocyte models, we compared fluxes in nRCM, aRCM and HL1 cells. We additionally investigated the effect of aRCM culturing on FAO fluxes. The FAO rate in freshly isolated aRCM amounted to 28 pmol/mg protein per min. This is an underestimation, since we previously showed that ^14^CO_2_ production from labeled palmitate was not detectable within the first 10 minute time-period of the assay^2^ (supplementary note 5). Nevertheless, these values are comparable to the palmitate oxidation rates measured in *in vivo* animals and in isolated perfused hearts using ^14^C/^3^H/^13^C techniques (0.2–1.3 μmol/g dry weight per min) summarized in ref^6^ (supplementary note 6).

After culturing cells remained viable and morphology was maintained (Fig. 1B). Cultured aRCM showed 3-fold lower FAO flux when compared to freshly isolated aRCM and 3-fold higher FAO flux as compared to either HL1 cells or nRCM (Fig. 1C). These data are in line with the acknowledged glucose preference over fatty acid substrates of both the nRCM and HL1 cells. Additionally, our data are in agreement with recent data of Mdaki *et al*.^7^ who showed ~2.5-fold higher FAO rates in cardiomyocytes of 3, 10 and 52–78 week old rats as compared to nRCM, as estimated by oxygen consumption rate.

### Mitochondrial content is maintained in adult rat cardiomyocytes after 2 days in culture

To investigate whether the different levels of FAO flux between different cardiomyocyte models could be explained by changes in expression of proteins constituting the electron transport chain, we measured protein expression levels of mitochondrial oxidative phosphorylation complexes (OXPHOS). After correcting for protein amount, fresh and cultured aRCM showed higher expression of OXPHOS as compared to nRCM and HL1 cells (Fig. 1D,E and Supplemental Figure S1). This is consistent with the bigger reliance on anaerobic lactate oxidation and glycolysis for energy production in both the nRCM and HL1 cells, as compared to aRCM. Interestingly, there was no difference in total OXPHOS levels between cultured and fresh aRCMs (Fig. 1D,E), while FAO flux was significantly higher in the latter. Additionally, the 8-fold lower FAO rate of the nRCM and HL1 cells vs fresh aRCM, though not statistically significant for nRCM, does not proportionally relate to the relative difference in OXPHOS levels (~1.5-fold lower). Therefore, mitochondrial OXPHOS levels do not predict FAO flux in intact cells.

### Short- or long-term incubation with oligomycin and the PPARα-ligand WY-14,643 increase and rotenone decreases FAO flux in cultured adult rat cardiomyocytes

**Figure 2:**
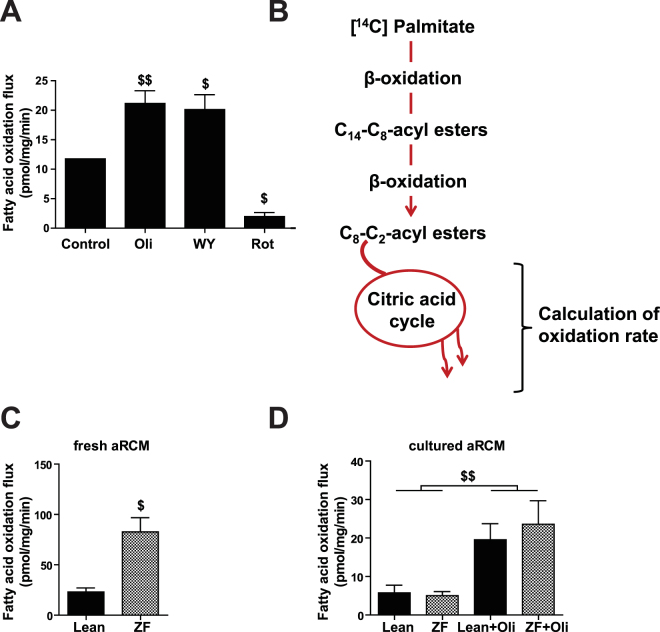
Modulation of FAO flux in aRCM. All aRCM were cultured in laminin-coated plates for 48 h; (**A**) FAO flux in untreated (control) adult rat cardiomyocytes (aRCM), or stimulated for 30 min with 5 μM oligomycin (Oli), or 3 μM rotenone (Rot), or 48 h with 10 μM of WY-14,643 (WY). Results are presented as mean ± SEM; ^$^p < 0.05; ^$$^p < 0.01; ^$$$^p < 0.001 vs control (n = 17, 6, 2, 3). (**B**) Different stages of ^14^C-palmitate oxidation (adapted from^17^); (**C**) FAO flux in untreated fresh aRCM isolated from lean or zucker fatty (ZF) rats, n = 3 per group, ^$^p < 0.05 vs lean; (**D**) FAO flux in 48 hours cultured aRCM from lean or ZF rats untreated or stimulated for 30 min with 5 μM oligomycin (Oli), n = 3 per group, ^$$^p < 0.01 oligomycin vs untreated. FAO fluxes are calculated from the amount of radiolabeled palmitate that is converted into ^14^CO_2_.

To further validate our FAO protocol, we used established short- and long-term stimulatory and inhibitory conditions (Fig. 2A,B). For this, aRCM were incubated with oligomycin, WY-14,643, or rotenone. Oligomycin increases the AMP/ATP ratio which activates AMPK thereby leading to increased FAO (supplementary note 7). Indeed, consistent with our previous data in freshly isolated aRCM^8,9^, we observed that short-term stimulation of cultured aRCM with oligomycin results in a 2-fold increase in FAO rate (p < 0.05).

WY-14,643 is a potent and widely used PPARα activator. The compound was previously shown to electively increase the expression of genes involved in cardiac FA metabolism in both nRCM^10^ and aRCM^11,12^. In nRCM it was shown to enhance FAO rate by 1.5-fold, both when measured as ^14^CO_2_ production^10^ or O_2_ consumption rate^13^. We here confirm the up-regulatory effect of PPARα-ligand WY-14,643 on the FA metabolism genes ucp2 (p < 0.05) and acsl1 (p < 0.05) and a trend to increased acox1 (p = 0.07) and cd36 (p = 0.07) in cultured aRCM (Supplemental Figure S2). In addition, we show that in aRCM, prior stimulation with WY-14,643 for 48 hours results in a 1.7-fold increase in FAO rate as compared to non-treated cells (control) (p < 0.05).

Rotenone is an inhibitor of OXPHOS complex I and is therefore expected to inhibit FAO flux. Indeed, we observed that short-term addition of rotenone led to a 6-fold decrease in FAO rate (p < 0.05) as compared to non-treated aRCM.

### Cardiomyocytes of Zucker Fatty rats present metabolic flexibility upon culturing

The obese Zucker Fatty (ZF) rat is a well-established genetic model of pre-diabetes^14^. Here we show that FAO rate is 3.6-fold higher in freshly isolated aRCM from ZF rats as compared to lean control Zucker Lean (ZL) rats (Fig. 2C). These findings are in line with the general view that FAO is increased in the early diabetic or obese heart^15,16^. Interestingly, the metabolic phenotype of the ZF aRCM on FAO fully disappeared after 48 hours of culturing, and normalized to the level of aRCM from ZL (Fig. 2D). The cardiomyocytes of the ZF rats did however show a similar 4-fold response to oligomycin-stimulation after culturing as cardiomyocytes of lean controls (Fig. 2D). These data indicate that the isolated cardiomyocytes of these pre-diabetic animals are still metabolically flexible and therefore able to adapt to environmental changes (i.e., cell medium) and stimuli. This suggests that the systemic environment in the pre-diabetic animals is mainly responsible for the metabolically altered phenotype in these cells.

Taken together, our data show that the presented protocol allows the monitoring of changes in FAO flux in cultured aRCM in response to both short- and long-term stimulatory as well as inhibitory conditions.

## Conclusion

This protocol describes a broadly applicable, easy-to-perform method for direct measurement of FAO fluxes in living cardiomyocytes, which does not require high instrument costs. In addition, by utilizing intact cells we avoid potential artefacts caused by mitochondrial isolation or cell permeabilization. Importantly, the latter assays do not take into account the regulation at the level of uptake and initial metabolic conversion of fatty acids, whereas our assay provides an integral measurement involving all steps: from uptake at sarcolemma to mitochondrial CO_2_ production. A great advantage of this method is that it analyzes the metabolic fate of palmitate as specific carbon source. Moreover, this protocol allows the investigation of the effects of long-term pharmacological, pathophysiological and/or genetic manipulations on FAO. We utilized chemical and pharmacological treatments in order to modulate the FAO flux at different cellular signalling levels. Short-term incubation of cardiomyocytes with the mitochondrial F_1_F_0_-ATP synthase inhibitor oligomycin and the mitochondrial complex I inhibitor rotenone resulted in significant up- and down-regulation of FAO flux, respectively. Furthermore, increasing FAO capacity at the gene level via prior incubation with the PPARα ligand WY-14,643, indeed boosted FAO flux in cultured aRCM. Finally, we demonstrate that our protocol allows measuring differences in FAO flux in freshly isolated cardiomyocytes from disease models.

Our data indicate that the commonly used nRCM and HL1 cardiomyocytes display FAO rates that are an order of magnitude lower than that of cultured primary adult rat cardiomyocytes. Caution should therefore be taken when using either neonatal cells or immortalized cell lines to study the impact of FA supply on the pathological and physiological mechanisms underlying the development of cardiac diseases such as heart failure. Furthermore, we conclude that OXPHOS protein content is not a good measure for FAO flux. Taken together, we have developed a straightforward method to measure FAO flux in living primary adult cardiomyocytes that is particularly suitable when using *in vitro* cardiac disease models including pharmacological, pathophysiological and/or genetic manipulations of choice.

## Electronic supplementary material

Supplementary information
