# Supplementary material for: Assessing fatty acid oxidation flux in rodent cardiomyocyte models
Source: Sci Rep. 2018 Jan 24;8:1505. doi: 10.1038/s41598-018-19478-9 (PMC5784119; doi:10.1038/s41598-018-19478-9)
Supplement: Supplementary file 1 — Supplementary information [file 41598_2018_19478_MOESM1_ESM.pdf]

Supplementary Information

Assessing fatty acid oxidation flux in rodent cardiomyocyte models

M. Rech[1], J.J.F.P. Luiken[2], J.F.C. Glatz[2], M. van Bilsen[1], B. Schroen[1,3], M. Nabben[2,3#]

[1] Department of Cardiology, CARIM School for Cardiovascular Diseases, Maastricht University, Maastricht, The Netherlands

[2] Department of Genetics and Cell Biology, CARIM School for Cardiovascular Diseases, Maastricht University, Maastricht, The Netherlands

[3] Co-senior author

[#] **Corresponding author:**
M. Nabben
Department of Genetics and Cell Biology
CARIM School for Cardiovascular Diseases, Maastricht University
P.O. Box 616, 6200 MD Maastricht, The Netherlands
e-mail: m.nabben@maastrichtuniversity.nl

Supplementary materials and methods

Animals

Male Lewis rats (300-450 g), 12-week-old male Zucker fatty and lean rats (Zuc-$Lepr^{fa}$ fa/fa and ZUC-$Lepr^{fa}$ fa/+, respectively) and 3-day-old Wistar neonatal rats were purchased from Charles River Laboratories (The Netherlands) and used for cardiomyocyte isolation. The animals were housed in a controlled environment (21–22°C) with a 12:12 hour light/dark cycle and free access to food and water.

Chemicals and media

For isolation and culturing, the following chemicals were used: Medium M199 (Gibco #31153); gelatin type B (Sigma G-9382); DMEM (#42430); horse serum (Gibco #16050); newborn calf serum (Sera-Lab, Sussex, UK); antibiotics (1% penicillin/streptomycin, Gibco BRL). aRCM medium consisted of M199 supplemented with 5 mM creatine, 3.2 mM carnitine, 3.1 mM taurine, 20 μM palmitate and 1% penicillin/streptomycin. nRCM medium consisted of a 4:1 mixture of DMEM and M199 supplemented with 10% horse serum, 5% newborn calf serum, and 1% penicillin/streptomycin and 0.5% gentamicin. Bovine serum albumin (fraction V fatty acid free), dependent on the application, was derived from MP Biomedicals (Irvine, USA) (for cell isolation and incubation purposes), or Sigma (St. Louis, MO) (other purposes).

For preparation of the ^{14}C-labeled palmitate-albumin complex and for the fatty acid oxidation assay the following chemicals were used: [1-^{14}C]palmitate (GE Healthcare, Little Chalfont, UK); laminin, oligomycin, phenformin and sodium azide from (Sigma, Saint Louis, USA); collagenase type II (Worthington, Freehold, USA); WY-14,643 (Enzo Bioscience, Antwerpen, Belgium); Optifluor solution (PerkinElmer, USA); BCA protein assay Kit (Pierce, USA).

Krebs Ringer (MKR) 1x solution was made from a 10x stock solution consisting of 1.17 M NaCL, 26 mM KCL, 12 mM KH_2PO_4, 12 mM $MgSO_4 * 7H_2O$, 100 mM $NaHCO_3$ and 100 mM HEPES, pH 7.55.

Preparation of the ^{14}C-labeled palmitate-albumin complex

Stock [l-^{14}C]palmitate label was prepared as described previously [1]. Briefly, [l-^{14}C]palmitate (50 μCi) and unlabeled palmitate (90 μmol), both dissolved in 10 mL 100% ethanol, were mixed with 10 mL distilled water containing 135 μmol KOH (i.e., 1.5 times the amount of palmitate on molar basis). After evaporation of ethanol at 45°C under nitrogen gassing, and re-adjusting the volume to 10 mL with distilled water, the KOH-palmitate solution was slowly added to 40 mL of a pre-warmed 1x modified Krebs Ringer (MKR) solution supplemented with 1.0 g fatty acid-free BSA

and CaCl2 (1.0 mM final) solution. The solution was then centrifuged (10 min at 2500 x *g*) and stored in 2 mL aliquots at -20°C. The resulting [l-^{14}C]palmitate stock solution consists of 1.8 mM [l-^{14}C]palmitate bound to 0.3 mM BSA (molar ratio 6:1).

Western blotting

Western blot analysis of OXPHOS protein was performed in cardiomyocyte lysates. Proteins (20 µg per sample) were separated by SDS-PAGE on 4-12% Bis-Tris Criterion XT precast gels (Biorad, California, USA). Proteins were transferred on nitrocellulose membrane (0.45 µm pore size, Millipore, Billerica, USA). The membrane was blocked in 5% milk in tris-buffered saline with 0,1% Tween for 1 hour at room temperature, incubated with total OXPHOS rodent WB antibody cocktail (1:1000, Abcam, Cambrige, UK) overnight at 4°C with gentle shaking, rinsed with tris-buffered saline with 0,1% Tween. Subsequently the membrane was incubated with secondary rabbit anti-mouse antibody (1:15000, DakoCytomation, Glostrup, Denmark) for 1hour at room temperature with gentle shaking. The membrane was washed and protein bands were visualized using enhanced chemiluminescence (Biorad, California, USA) and immunoblot intensities were analyzed by densitometry with the software Image Studio Lite (Westburg, Leusden, Netherlands).

RNA analysis

Total RNA was isolated from aRCM with the miRVana microRNA isolation kit (Ambion, Austin, TX). A miScript PCR system (Qiagen) was used for cDNA synthesis. SYBR Green® quantitative PCR was performed on a Bio-Rad iCycler (Hercules, CA) to determine gene expression levels of ucp2, acsl1, acox1, cd36 genes with the respective primers: Ucp2 Fw: GCACTCCTGTGTTCTCCTGT, Rev GAAAATGTCTGGGAGACGAA; Acsl1 Fw: GAGGGTGAGGTGTGTGTGAA, Rev: CAGCTGTTCTTGCTGGGTCT; Acox1 Fw: CGTGCAGCCAGATTGGTAGAA, Rev: CTACTTCCTTGCTCTTCCTGTGACT; cd36 Fw: TTTCCTCTGACATTTGCAGGTCTA, Rev: AAAGGCGTTGGCTGGAAGAA. Cyclophilin A was used for normalization; Fw CAAATGCTGGACCAAACACAA, Rev: TTCACCTTCCCAAAGACCACAT

Notes

1. Extra care should be taken into account when detaching the cardiomyocytes from the culture plate. Since trypsin, at concentrations >0.05%, is known to inhibit FA uptake[2] it cannot be used for the purpose of this assay.

2. In order to be included in the experiment it was required that >80% of the aRCM were rod-shaped and excluded trypan blue.

3. The corresponding BSA-palmitate ratio is 0.75, thus 2x below the K_M for the uptake process [2]. Given that the trans-sarcolemmal uptake is the rate controlling step in the total oxidation process of FA, this indicates that under these conditions, changes in FAO flux can be sensitively detected.

4. A zero time control (ZTC) condition is a correction for the background signal, and therefore serves as a negative control for the assay. There should be at least one incubation per each experiment. In the ZTC, the stop solution is added just before addition of DLOx. When performing the calculation of FAO, the counts of this ZTC have to be substracted from the radioactive counts of each incubation.

5. We have previously shown that $^{14}CO_2$ production from labeled palmitate was detectable after 10 minutes and increased linearly with time for at least 2 hours[2], indicating that oxidation, as measured during our current 30-min protocol, only takes place during the 10 to 30 min time period.

6. Calculation: 28 pmol/mg protein/min equals ~5.0 nmol/g wet weight per min (as 1 gram wet weight equals ~178 mg protein [2]). After correcting for the administered palmitate concentration (e.g. 500 µM is used in O'Donnell's [3] versus 100 µM in our study) and considering that *in vivo* cells are contracting and thus have higher FAO rates than *ex vivo* cells (i.e. 4 Hz stimulation of aRCM led to a 2.8 fold upregulation in FAO[4]), the FAO value in our study compares to ~70 nmol / g wet weight / min. This is ~0.56 µmol/g dry weight per min (as 1 gram dry weight equals 8 gram wet weight [5]).

7. Oligomycin is a specific inhibitor of mitochondrial F_1F_0-ATP synthase and is often shown to inhibit FAO. However, Ylitalo et al.[6] showed that in isolated rat heart mitochondria there is a range of oligomycin concentrations at which there are elevated intracellular AMP levels, without any inhibitory actions on oxygen consumption rates. The increased intracellular AMP/ATP ratio then leads to AMPK activation, phosphorylation of ACC and subsequent de-inhibition of CPT-I, thereby leading to increased FAO.

Supplementary figures

Figure S1.

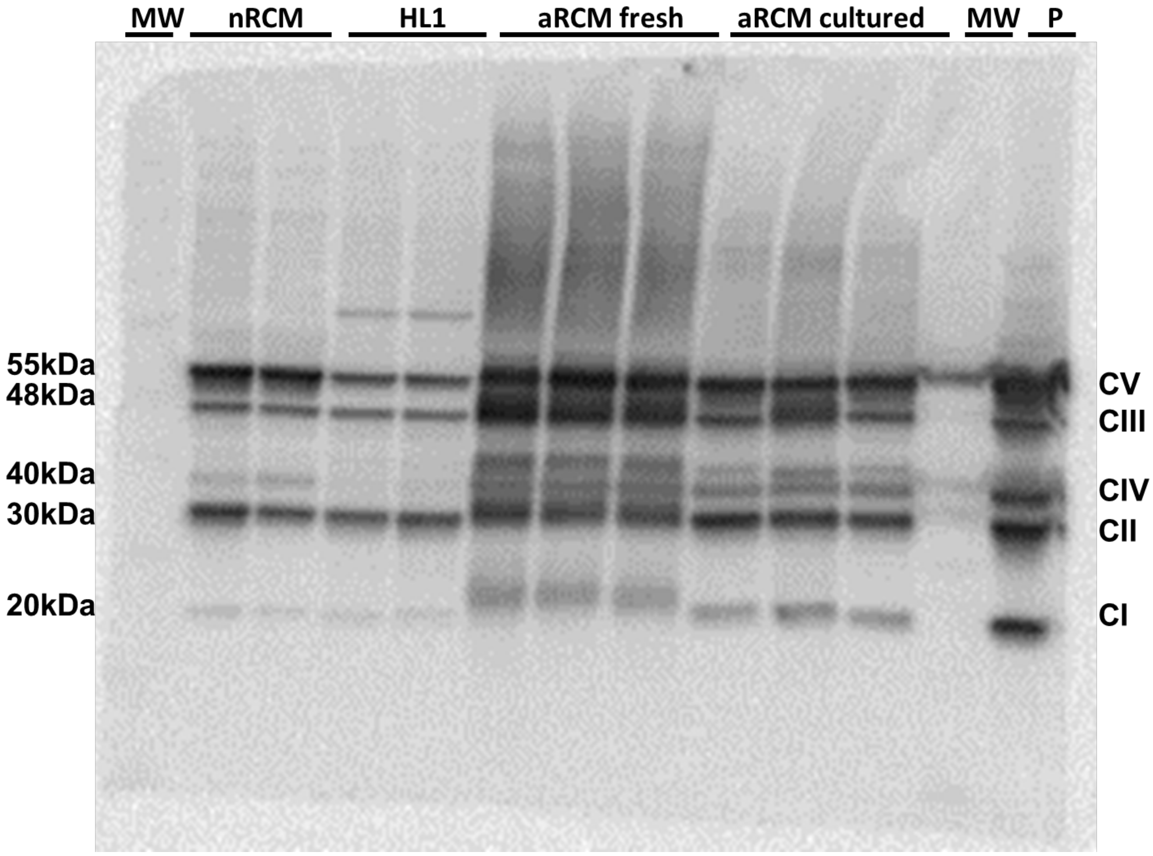

Figure S1. Full-length blot of OXPHOS protein that is shown in the main article in figure 1D. Western blot analysis was performed in lysates of neonatal rat cardiomyocytes (nRCM), HL1 cardiomyocytes (HL1), and fresh and cultured adult rat cardiomyocytes (aRCM). The lane where the molecular weight marker was loaded is marked with MW. Rat heart mitochondria served as positive control (P).

Figure S2.

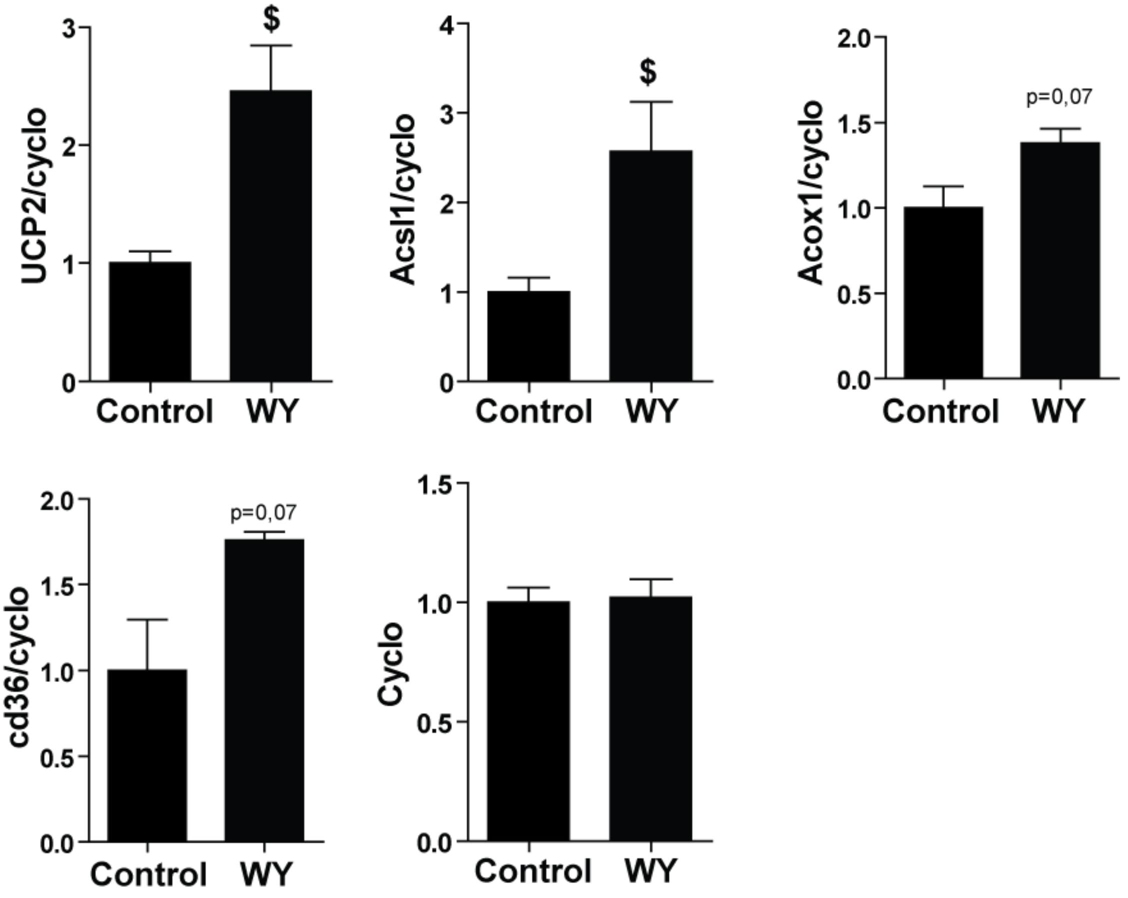

Figure S2. Gene expression analysis of untreated (control) adult rat cardiomyocytes (aRCM), or stimulated for 48h with 10 μM of WY-14,643 compound (WY); *ucp2*, mitochondrial uncoupling protein 2; *acsl1*, acyl-CoA synthetase; *acox1*, acyl-Coenzyme A oxidase 1; *cd36*, cluster of differentiation 36; gene expression data are normalized by the housekeeping'gene *cyclo*, cyclophilin A; $ p<0.05 vs Control.

References

1 Luiken, J. J. F. P. *et al. Methods of Molecular Biology - AMPK methods and protocols*. (Springer, accepted contribution).

2 Luiken, J. J., van Nieuwenhoven, F. A., America, G., van der Vusse, G. J. & Glatz, J. F. Uptake and metabolism of palmitate by isolated cardiac myocytes from adult rats: involvement of sarcolemmal proteins. *Journal of lipid research* **38**, 745-758 (1997).

3 O'Donnell, J. M., Alpert, N. M., White, L. T. & Lewandowski, E. D. Coupling of mitochondrial fatty acid uptake to oxidative flux in the intact heart. *Biophysical journal* **82**, 11-18, doi:10.1016/s0006-3495(02)75369-1 (2002).

4 Luiken, J. J., Willems, J., van der Vusse, G. J. & Glatz, J. F. Electrostimulation enhances FAT/CD36-mediated long-chain fatty acid uptake by isolated rat cardiac myocytes. *American journal of physiology. Endocrinology and metabolism* **281**, E704-712 (2001).

5 Heather, L. C. *et al.* Fatty acid transporter levels and palmitate oxidation rate correlate with ejection fraction in the infarcted rat heart. *Cardiovascular research* **72**, 430-437, doi:10.1016/j.cardiores.2006.08.020 (2006).

6 Ylitalo, K. *et al.* Reversible ischemic inhibition of F(1)F(0)-ATPase in rat and human myocardium. *Biochimica et biophysica acta* **1504**, 329-339 (2001).
